# Cutaneous vasculitis; An algorithmic approach to diagnosis

**DOI:** 10.3389/fmed.2022.1012554

**Published:** 2022-09-21

**Authors:** Erkan Alpsoy

**Affiliations:** Department of Dermatology and Venereology, School of Medicine, Akdeniz University, Antalya, Turkey

**Keywords:** vasculitis, leukocytoclastic, cutaneous, algorithms, IgA vasculitis

## Abstract

Vasculitides, characterized by inflammation and damage of blood vessels, encompass a broad spectrum of diseases. They can occur with different pathophysiological mechanisms and have a rich clinical heterogeneity depending on the vessel diameters they affect. Vasculitides may also present with a broad spectrum of severity, ranging from a mild self-limiting to a potentially life-threatening disease. The high prevalence of skin involvement in vasculitis, visible character and, finally, the easy accessibility of the skin for both physical examination and biopsy offers important advantages for prompt disease recognition and diagnosis. Thus, dermatologists are privileged to diagnose the disease earlier and more effectively than any other discipline. As a consequence, a detailed clinical and histopathological evaluation of the skin is one of the most critical steps in diagnosing vasculitis. Besides obtaining a good medical history, laboratory and radiological evaluation methods are used in the diagnosis. In this review, a practical and algorithmic approach is aimed to assist in the diagnosis of vasculitis. However, this approach should not be seen as strict rules. This stepwise algorithmic diagnostic approach for vasculitis was developed by combining the current literature knowledge and the author's experience in this field to provide a rational framework for selecting the most appropriate among various diagnostic approaches.

## Introduction

Vasculitis refers to a broad and heterogeneous disease spectrum characterized by inflammation and damage of the blood vessel. It may occur in any organ of the body. When skin vessels are affected, the term cutaneous vasculitis is used. In systemic vasculitis, blood vessels of at least one organ are affected in addition to the skin. Of note, besides being a component of systemic vasculitis, including the skin, cutaneous vasculitis can be a skin-limited or skin-dominant expression or variant of systemic vasculitis. Finally, it may be a single-organ vasculitis of the skin (1). The skin is one of the most frequently affected organs in vasculitis, and small-vessel vasculitis of the skin is the most common vasculitis dermatologists encounter in their clinical practice (2). While the disease affects both genders equally, its frequency increases with age at diagnosis. Cutaneous vasculitis may present at any age, but it is more common in adults than children. The clinical course of the disease is usually self-limiting in children, and IgA vasculitis is the most common vasculitis in this age group. Idiopathic etiology is more prominent in adults and infectious etiology in children. Compared with children, underlying systemic vasculitis, connective tissue disease, or malignancy are more common in adult patients (3, 4).

The skin is a visible and easily accessible organ for physical examination and biopsy, providing significant advantages over other organs' vasculitides in diagnosing vasculitis. Accurate identification of cutaneous lesions and a good histopathological examination with biopsy that can be easily and safely taken from the skin can provide precious information for the recognition and diagnosis (5). This review overviews the current literature knowledge regarding the diagnosis of vasculitis by combining the author's experience in this field and proposes a stepwise algorithmic diagnostic approach to enable clinicians to rationalize the selection of the most appropriate diagnostic approach.

## Clinical presentation

Vasculitis may occur with different pathophysiological mechanisms and may cause different clinics depending on the vessel's diameter (1, 2, 5). In addition, some vasculitis patients with initially non-severe mild symptoms and limited organ involvement may progress in severity over several days to weeks, affecting multiple organs. In summary, patients with vasculitis may present with a broad spectrum of severity ranging from a mild self-limiting disease to a potentially life-threatening one (6).

Physicians should carefully consider some critical steps in clinical practice when diagnosing vasculitis. First, skin manifestations should be morphologically and histologically compatible with vasculitis. Then, the underlying etiological cause/s should be investigated. For this purpose, a good medical history, possible triggering factors, especially recently introduced drugs and recent infections, should be questioned in detail, and finally, extracutaneous involvement should be evaluated (2, 5).

How does vasculitis suspicion begin in the clinic? The first question to be answered in the clinical examination is whether the lesions are compatible with vasculitis. Palpable purpura is the main dermatological finding of small-vessel vasculitis. The suspicion of vasculitis increases if the palpable purpura is symmetrically located on the lower extremities. Purpura often develops in groups and may be accompanied by pain, burning, and itching (2, 6). Although palpable purpura is the most critical elementary lesion in the vasculitis spectrum, a wide range of elementary lesions can be obtained. Other skin findings of vasculitis include urticarial papules, plaques, nodules, vesicles, bullae, pustules, ulcers, and target-like lesions (5, 7). There is almost no disease-specific primary lesion or organ involvement (7).

The clinical appearance in patients with vasculitis is closely related to the diameter of the involved vessel. For this reason, vasculitis is classified according to the vessel diameter. Ulcers, nodules, pitted scars, white atrophy and livedo racemosa indicate the deep plexus, and medium-sized vessel involvement at the dermohypodermal junction. On the other hand, edematous papule, plaque, and palpable or non-palpable purpura occur due to the involvement of small vessels within the superficial or subpapillary plexus (5, 7). It should be kept in mind that a proper clinical examination may help limit the diagnosis to a specific area by eliminating many diseases within the vasculitis spectrum.

## Skin biopsy: Number, timing, depth, and location

When the suspicion of vasculitis occurs, the first step should be to confirm the diagnosis by skin biopsy. Histopathology is the “gold standard” for the diagnosis of cutaneous vasculitis. An appropriate sampling of skin biopsy is crucial to increase its diagnostic value. In this sense, **one** of the first questions to be considered is the number of skin samples biopsied. Two separate skin biopsies are recommended. In addition to the skin biopsy for routine evaluation with a light microscope, taking a second skin biopsy for direct immunofluorescence (DIF) is also recommended. Vasculitis is a dynamic process, and the inflammatory infiltrates change over time. Therefore, the timing of the skin biopsy is also critical. Typical histopathological changes for vasculitis develop 24–48 h after the appearance of lesions. Biopsy should be performed at the appropriate depth. Deep punch biopsy or excisional biopsy reaching the subcutis is recommended. As a result, small- and medium-sized vessel vasculitides of the skin can only be evaluated with an appropriate biopsy (2, 6).

Identifying the most appropriate area for the skin biopsy is another critical step in diagnosing vasculitis. Lesional skin should be preferred. Biopsy should be performed from purpuric papules for a light microscope and from a blanchable macule for DIF to detect immunoglobulin deposition in the vascular wall. Diascopy helps select the appropriate lesion for biopsy. Blanchable areas by diascopy show inflammation (erythema), while non-blanchable areas correspond to purpura (erythrocyte extravasation). It should be kept in mind that negative DIF results may be obtained in lesions older than 48 hours due to the rapid destruction of immune accumulations. Therefore, the diagnosis of vasculitis should not be based solely on positive or negative DIF findings. Instead, it should be interpreted together with history, clinical, histopathological, and other laboratory findings (2, 5, 7).

When histopathology is consistent with vasculitis, leukocytoclastic vasculitis is the most commonly observed histopathological appearance. It is distinguished by neutrophilic infiltration of the vessel walls, nuclear dust, fibrinoid degeneration, endothelial edema, and erythrocyte extravasation around postcapillary venules. In older lesions, neutrophils decrease, and mononuclear cells, particularly lymphocytes, predominate. Notably, granulomatous vasculitis was reported more frequently with systemic vasculitis, especially in lymphoproliferative diseases (8). On the other hand, lymphocytic vasculitis is more common in connective tissue diseases, viral infections and drug eruptions (2).

## Etiological examination

The following step after the diagnosis of vasculitis should be the investigation of etiological causes. According to the current literature, the etiologic factor cannot be detected in approximately half of the vasculitis cases (idiopathic etiology; 45–55%). Infection, especially streptococcus pyogenes, Hepatitis B and C virus, and HIV can be detected in 15–20% of cases (2, 9, 10). Although mostly limited to isolated reports, COVID-19-associated and anti-SARS-CoV-2-vaccine-associated cutaneous vasculitis has been reported during the pandemic (11, 12). Inflammatory diseases (inflammatory bowel diseases, cryoglobulinemia type 2 and 3, antineutrophilic cytoplasmic antibody (ANCA)-related vasculitis and Behçet's disease) and connective tissue diseases (systemic lupus erythematosus, rheumatoid arthritis, sjögren syndrome) are the etiologic factors in 15–20% of the cases. Drugs (β-lactam antibiotics, sulfa preparations, minocycline, non-steroidal anti-inflammatory drugs, granulocyte-macrophage colony-stimulating factor, propylthiouracil, tumor necrosis factor (TNF)-alpha inhibitors, levamisole loaded cocaine, etc.) in ~10–15% of cases, and malignancy in 5% of cases (hematologic malignancies, solid organ cancers, etc.,) play a role in the development of vasculitis (2, 9, 10).

All patients with vasculitis are evaluated as a laboratory to detect the underlying cause and possible systemic involvement. There is no agreement on a standard screening protocol yet. Nevertheless, the primary goal should be to identify the underlying cause and severity of organ/s involvement based on clinical signs and symptoms. All patients with suspected vasculitis should be evaluated for complete blood count, creatinine, sedimentation rate, liver function tests, urinalysis, and chest X-ray. If the patient has symptoms such as fever, weight loss, fatigue, arthralgia, myalgia, hematuria, abdominal pain, blood in the stool, numbness, paresthesia, neuralgia, dyspnea, chest pain, cough, hemoptysis, sinusitis, more detailed examinations are required. A detailed etiological investigation is also conducted in patients with chronic and recurrent vasculitis whose etiology cannot be determined in previous episodes of vasculitis. In addition to the above, more extensive tests including ASO, throat culture, CRP, HBV, HCV, ANA, Anti-ds DNA, Anti Ro, Anti-La antibodies, RF, CCP, HIV, C3, C4, ANCA, cryoglobulin, immune electrophoresis, peripheral smear, chest X-ray, fecal occult blood, etc. should be performed (2, 7).

## Stepwise algorithmic approach in the diagnosis of cutaneous vasculitis

**Figure 1:**
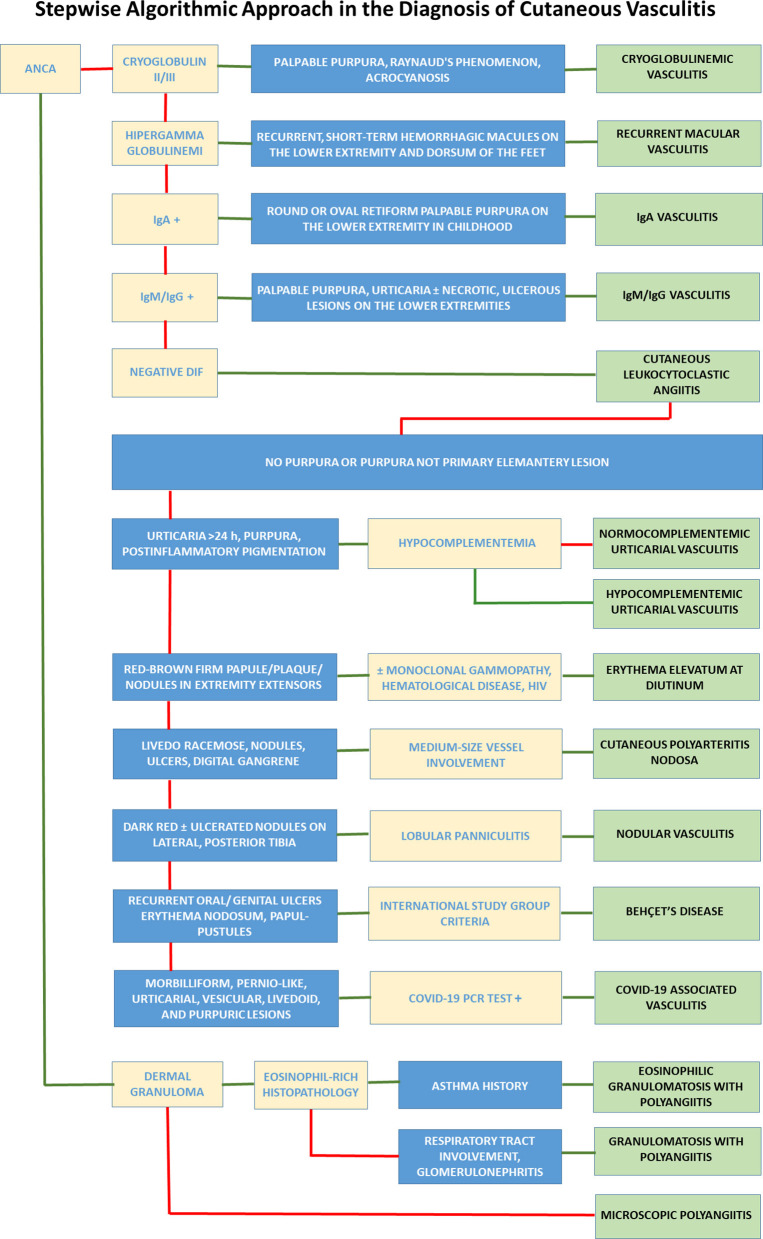
Stepwise, algorithmic diagnosis of vasculitis. In the flowchart, all disease diagnoses are placed one under the other in the far right column. Algorithms start from the boxes in the upper left corner. The green arrow means “yes”, and the red arrow means “no”. ANCA, antineutrophilic cytoplasmic antibody; Ig, Immunoglobulin; DIF, direct immunofluorescence.

Medical history, clinical, histopathological, radiological, and other laboratory evaluations are used to diagnose vasculitis. It is challenging to develop a stepwise algorithmic diagnostic approach for all vasculitis. The algorithmic approach given here is intended to aid in the diagnosis. However, this approach should not be considered as strict rules to be followed. The following algorithmic diagnostic approach was developed by combining the current literature knowledge and the author's experience in this field. It should be kept in mind that vasculitis, which initially seems limited to the skin, may also develop systemic involvement over time. Stepwise, algorithmic diagnosis of vasculitis is summarized in Figure 1.

In ANCA negative cases, if cryoglobulin 2 or 3 are positive in DIF and clinically palpable purpura, Raynaud's phenomenon, and acrocyanosis are present, cryoglobulinemic vasculitis should be considered in the first place. Types 2 and 3 cryoglobulins are called mixed cryoglobulinemia and may be associated with B-cell lymphoproliferative diseases, autoimmune diseases, and infection. It can affect the peripheral nerves and kidneys as well as the skin. Type 1 causes Reynaud's phenomenon with vascular occlusion, ulcers, pain and oedema in the extremities, or hyperviscosity syndrome (1).

With hypergammaglobulinemia, if there are recurrent and short-term hemorrhagic macules occurring mainly on the lower extremities and dorsum of the feet, recurrent macular vasculitis should be the diagnosis. Polyclonal hypergammaglobulinemia, primarily composed of IgG, is the hallmark of the disease. It usually has a good prognosis; however, sometimes, it can be a sign of underlying connective tissue disease or hematological malignancy (13).

In the presence of IgA deposition in DIF and clinically round or oval and retiform palpable purpura, sometimes accompanied by hemorrhagic vesicle/bulla, on the lower extremities, especially in a school-age child, IgA vasculitis should be considered. The other main clinical symptoms are arthritis, gastrointestinal bleeding or pain, and glomerulonephritis with mesangial IgA deposits (14). IgA vasculitis is more severe in adults. While joint and gastrointestinal involvement is more common in younger patients, severe purpura and glomerulonephritis are more common in older patients (15). IgA vasculitis is the most common vasculitis of childhood. It constitutes approximately 10% of cutaneous vasculitides (16, 17).

Similarly, the diagnosis of IgM/G vasculitis is achieved in patients who show IgM/G deposition instead of IgA deposition in DIF with a similar clinical presentation with palpable purpura, urticaria and sometimes necrotic/ulcerous lesions symmetrically located on the lower extremities (18).

If there is no immunoreacting in the vessel wall, and there are clinical and light microscopic findings similar to IgA vasculitis, the diagnosis of cutaneous leukocytoclastic angiitis is achieved. It is an isolated cutaneous vasculitis characterized by the involvement of post-capillary venules without systemic involvement. Thus, cutaneous leukocytoclastic angiitis is a diagnosis of exclusion. However, some systemic vasculitides (IgA vasculitis, granulomatosis with polyangiitis, microscopic polyangiitis) may initially present as cutaneous leukocytoclastic angiitis (17).

If the primary lesion is not purpura or the patient does not have purpura, the diseases summarized below should be considered first.

When urticarial papules and plaques persisting for more than 24–48 h are accompanied by purpura and postinflammatory hyperpigmentation, urticarial vasculitis should be considered. In these patients, when the complement level is within the normal range, the diagnosis is normocomplementemic vasculitis, and when the complement level is low, the diagnosis is hypocomplementemic vasculitis. While normocomplementemic urticarial vasculitis is often idiopathic, hypocomplementemic urticarial vasculitis represents systemic vasculitis, with various manifestations, mainly musculoskeletal (e.g., SLE, primary Sjögren's syndrome) and ocular involvement associated with anti-C1q antibodies (5, 19).

In the presence of red-brown firm papules, plaques and nodules on the extensor surfaces of the fingers, hands, elbows, ankles, and knees, erythema elevatum at diutinum are considered. It should be kept in mind that monoclonal gammopathy, hematological disease, and HIV may accompany these conditions (6, 20).

If the primary clinical lesions are livedo, especially livedo racemose, nodules, ulcers, digital gangrene in the absence of systemic involvement, and histopathologically medium-sized vessels in addition to small-sized vessels are involved, cutaneous polyarteritis nodosa should be considered. The lower extremities are frequently involved. Atrophy Blanche, Raynaud's phenomenon, plaques surrounded by inflammatory papulonodules may accompany (1, 6). Deficiency of Adenosine deaminase 2 (DADA2) is the first molecularly described monogenic vasculitis syndrome, caused by mutations in ADA2 gene, which encodes an extracellular enzyme acting as a monocyte differentiation factor. DADA2 has been defined as a clinical picture resembling polyarteritis nodosa, including livedo racemose, recurrent fever, and musculoskeletal complaints (21). Therefore, it can be considered in the differential diagnosis of cutaneous polyarteritis nodosa in selected cases.

If there are recurrent, dark red-violet, sometimes ulcerated nodules on the lateral and posterior surfaces of the tibia and lobular panniculitis are seen in light microscope examination, nodular vasculitis should be considered first. Nodular vasculitis may develop as an id reaction due to hypersensitivity to M. Tuberculosis, especially in endemic areas. In this case, it is called “erythema induratum bazin” (1).

Behçet's disease should be considered first in patients presenting with recurrent oral and genital ulcerations, erythema nodosum-like lesions and papulopustular lesions. Pustular lesions with a purpuric rim (pustular vasculitis) and superficial thrombophlebitis can be seen in the course of the disease (22). International Study Group criteria are the most widely used diagnostic criteria in diagnosis (23).

The cutaneous manifestations of Coronavirus disease 2019 (COVID-19) are significantly varied and include morbilliform, pernio-like, urticarial, vesicular, livedoid, and purpuric lesions. COVID-19-associated vasculitis most commonly presents as cutaneous small vessel vasculitis. However, its prevalence is lower (1.8%) than other dermatological manifestations. The clinical presentation ranges from classic, bilaterally symmetrical palpable purpura preferring the lower extremities to vesiculobullous, hemorrhagic, urticarial, or targetoid eruptions (24, 25). Therefore, in the presence of morbilliform, pernio-like, urticarial, vesicular, livedoid, and purpuric lesions together with COVID-19 PCR test positivity, COVID-19-associated vasculitis should be considered.

In ANCA-positive vasculitis, histopathology can be in the form of leukocytoclastic vasculitis, vasculitis of small arteries and arterioles, or granulomatous inflammation without vasculitis. Eosinophilic granulomatosis with polyangiitis (EGPA, formerly Churg-Strauss Syndrome) should be considered first if there is a history of asthma with eosinophil-rich histopathology in addition to granulomatous inflammation. Granulomatosis with polyangiitis (GPA, formerly Wegener's granulomatosis) should be considered in the presence of granulomatous inflammation without vasculitis in histopathology and upper and lower respiratory tract involvement and rapidly progressing glomerulonephritis. When these two diseases are excluded, the diagnosis should be microscopic polyangiitis (MPA) (17). In a large cohort including 1,184 patients with ANCA-associated vasculitides, cutaneous involvement were found more frequently in those with EGPA (47%) compared to GPA (34%) and MPA (28%). Petechiae/purpura (15%) in all types of ANCA-associated vasculitides was the most frequently reported skin manifestation, followed by painful skin lesions (8%) and maculopapular rash (8%). Allergic and nonspecific manifestations such as pruritus, urticaria, and maculopapular rash were significantly more common in EGPA patients than in GPA and MPA patients. Livedo reticularis and livedo racemosa were reported more frequently in MPA patients. Systemic involvement was more pronounced in patients with skin lesions, especially those with GPA and EGPA, than those without skin manifestations (26).

## Limitations and strengths

The presented algorithmic diagnostic approach reflects a standardized diagnostic approach limited to the author's knowledge and experience in this field and is not consensus-based. Moreover, evidence that the use of the diagnostic algorithm presented here improves patient outcomes is lacking. On the contrary, this diagnostic algorithm can be beneficial by highlighting the diagnostic steps and diseases not being immediately considered in clinical practice.

## Conclusions

Dermatologists are in a privileged position in the recognition and early diagnosis of cutaneous vasculitis because of the high prevalence of cutaneous involvement in vasculitis and the easy accessibility of the skin. In addition, the presence and/or spectrum of skin lesions can also be a predictive sign for diseases associated with severe systemic manifestations. Any type of skin lesions resembling vasculitis, including palpable or non-palpable purpura, edematous papule, plaque, ulcers, nodules, pitted scars, white atrophy, livedo racemosa, requires a skin biopsy which is the gold standard for the diagnosis of cutaneous vasculitis.

## Author contributions

Writing, acquisition of clinical data, conception, and design: EA. The author contributed to the article and approved the submitted version.

## Conflict of interest

The author declares that the research was conducted in the absence of any commercial or financial relationships that could be construed as a potential conflict of interest.

## Publisher's note

All claims expressed in this article are solely those of the authors and do not necessarily represent those of their affiliated organizations, or those of the publisher, the editors and the reviewers. Any product that may be evaluated in this article, or claim that may be made by its manufacturer, is not guaranteed or endorsed by the publisher.
